# Surgical approach for a refractory enterocutaneous fistula by combining laparoscopic surgery and a planned open conversion: a case report

**DOI:** 10.1186/s40792-024-01987-7

**Published:** 2024-08-14

**Authors:** Makoto Hasegawa, Takayuki Ogino, Yuki Sekido, Mitsunobu Takeda, Tsuyoshi Hata, Atsushi Hamabe, Norikatsu Miyoshi, Mamoru Uemura, Yuichiro Doki, Hidetoshi Eguchi

**Affiliations:** Department of Gastroenterological Surgery, Graduate School of Medicine, Osaka University, 2-2 Yamadaoka E-2, Suita, Osaka 565-0871 Japan

**Keywords:** Enterocutaneous fistula, Surgical approach, Adhesiolysis, Laparoscopic surgery, Open conversion

## Abstract

**Background:**

An enterocutaneous fistula (ECF) is defined as an abnormal communication between the gastrointestinal tract and skin. ECFs are rarely encountered in clinical practice, yet are frequently difficult to treat. Few reports exist regarding the surgical techniques for the treatment of an ECF. Therefore, we report a case of refractory ECF with concomitant severe adhesions, in which we performed combined laparoscopic adhesiolysis and planned open conversion.

**Case presentation:**

A 57-year-old female patient underwent a laparotomy for an ovarian cyst in her 20s. At 46 years, adhesiolysis without bowel resection was performed for adhesive small bowel obstruction (SBO). However, her symptoms did not improve. Eighteen days postoperatively, she underwent a reoperation and jejunostomy. An ECF developed post-reoperation; therefore, stoma closure and radical surgery for the ECF were planned. Due to the severe adhesions, only stoma closure was performed, based on intraoperative assessments. The patient was subsequently referred to our hospital. First, skin care around the fistula was provided during an outpatient visit. Appropriate sizing of the stoma pouch was performed, to improve erosions and ulcers. Thereafter, debridement of the perifistula skin and simple closure of the ECF outlet were attempted; however, the ECF recurred shortly thereafter. After 8 years of regular skin care, with the ECF remaining stable, however, manifesting as symptomatic SBO, she underwent laparoscopic adhesiolysis. This procedure was initiated in the epigastric region, where relatively fewer adhesions were anticipated. Post-open conversion, partial resection of the small intestine at four locations, including the fistula site, was performed. Postoperatively, jejunal edema and peristaltic dysfunction, due to narrowing of the superior mesenteric artery occurred. Regular drainage by percutaneous endoscopic gastrostomy was required. However, she improved and was discharged 3 months post-operatively. Three years post-operatively, the ECF and SBO did not recur.

**Conclusions:**

We reported a case of refractory ECF in which we were able to safely perform surgery, by combining laparoscopic adhesiolysis and a planned open conversion. Therefore, the surgical approach used in this case may be an option for securing a safe surgical field, while avoiding collateral damage.

## Background

An enterocutaneous fistula (ECF) is defined as an abnormal communication between the gastrointestinal tract and skin. ECFs are rarely encountered in clinical practice, yet are frequently difficult to treat. Largely, ECFs result as post-operative complications. Other causes include inflammatory bowel disease, malignancy, trauma, radiation injury, ischemia, tuberculosis, and diverticulitis [1].

Currently, no treatment guidelines exist for ECFs, with each institution utilizing distinct treatment protocols. However, the American Society for Parenteral and Enteral Nutrition (ASPEN) and Federación Latino Americana de Terapia Nutricional, Nutrición Clínica y Metabolismo (FELANPE) have issued guidelines regarding nutritional therapy for ECFs [2]. In most cases, the first choice of treatment is noninvasive. With no improvement, surgical intervention is subsequently performed. Less invasive treatments include negative pressure wound therapy [3–6], fibrin glue [7, 8], and endoscopic closure [9], with varying closure rates [10–13]. However, postoperative recurrence of ECFs are known to occur [10]. Cases of ECFs involve severe adhesions, due to intra-abdominal inflammation; and a history of multiple surgeries, requiring complicated techniques for adhesiolysis. Thus, selecting the correct approach for surgical treatment is challenging. Only a few reports exist on the surgical techniques for the treatment of ECFs.

Consequently, we report a case of refractory ECF with concomitant severe adhesions, in which we performed combined laparoscopic adhesiolysis and planned open conversion.

## Case presentation

**Fig. 1:**
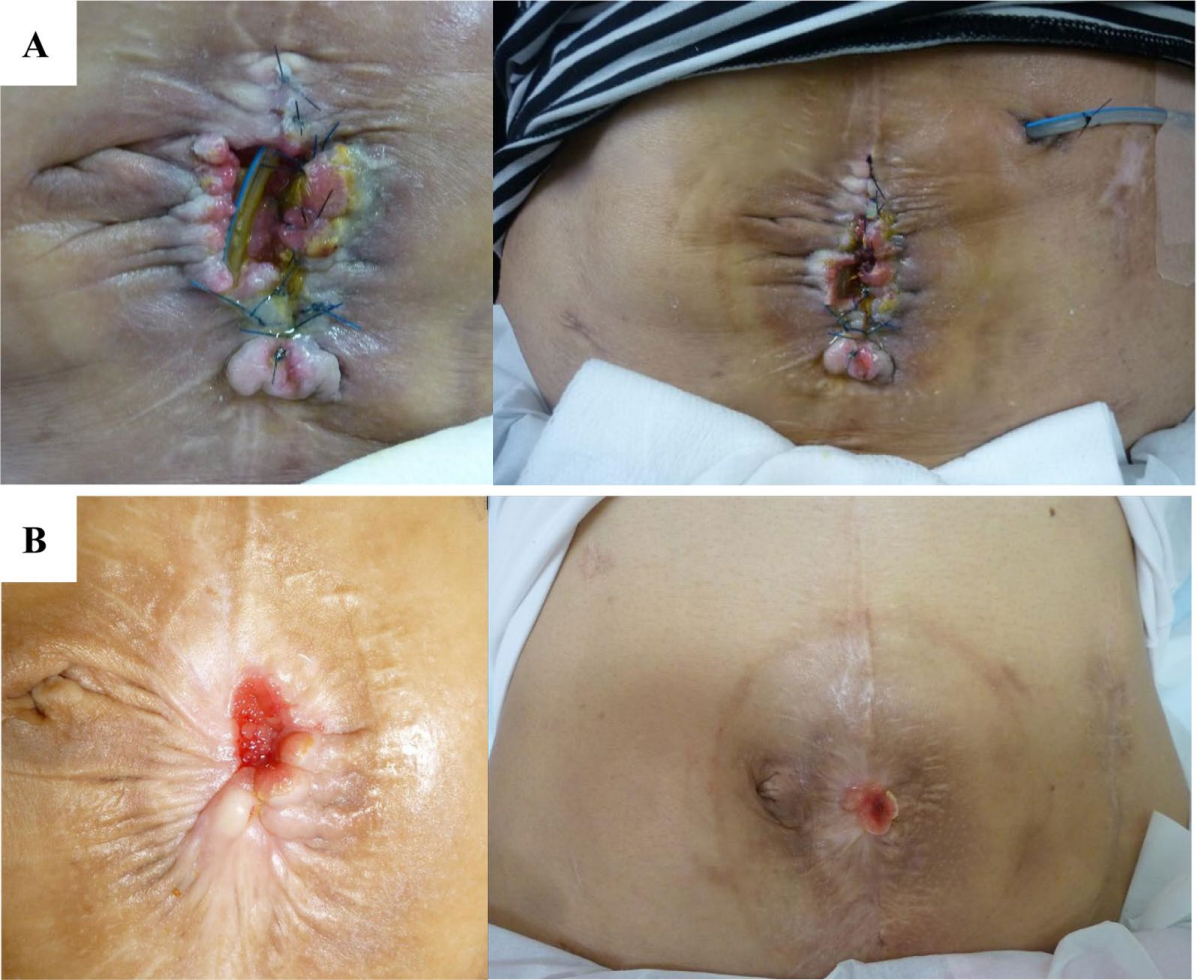
Enterocutaneous fistula (ECF) is depicted. **A** State of the ECF at the time of referral to our hospital is shown. **B** State of the ECF after skin care is shown

A 57-year-old female patient had previously undergone a laparotomy for an ovarian cyst in her 20s. At 46 years, due to adhesive small bowel obstruction (SBO), adhesiolysis without bowel resection was performed. However, her symptoms did not improve. Eighteen days postoperatively, a reoperation was performed for SBO; nonetheless, she only underwent a jejunostomy, due to the severe adhesions. Subsequently, 31 days after the primary surgery, leakage of intestinal fluid was observed from the laparotomy wound; and an ECF developed (Fig. 1A). Three months thereafter, resection of the ECF with stoma closure was planned. However, due to the severe adhesions, only stoma closure was performed, based on intraoperative assessments. Post-stoma closure, festering and ulceration of the peristomal skin worsened, resulting from leakage of the intestinal fluid from the ECF.

Consequently, as a result of difficult management, the patient was referred to our hospital. First, skin care around the fistula was performed, during an outpatient visit; the stoma pouch was appropriately sized with the use of skin protectants, to prevent contamination around the ECF and to improve skin erosions and ulcers (Fig. 1B). No other treatments such as negative pressure wound therapy were applied. Eighteen-month post-stoma closure, debridement of the perifistula skin and simple closure of the ECF outlet were attempted. Nevertheless, the ECF recurred shortly postoperatively. For the subsequent 8 years, the ECF remained stable with regular skin care. Regarding nutritional status upon referral, the albumin level was 3.5 g/dL, lymphocyte count was 1.0 × 10^3^/μL, hemoglobin level was 9.5 g/dL, and cholinesterase level was 189 U/L. Using magnesium oxide and kampo, such as Daikenchuto, and by encouraging oral intake, primarily of soft foods, these parameters improved to normal ranges approximately 4 months after referral to our hospital. The output of intestinal fluid from the ECF was controlled and did not exceed 600 mL/day. The patient experienced symptoms of SBO approximately four times a year. However, she persistently refused to undergo surgery.

**Fig. 2:**
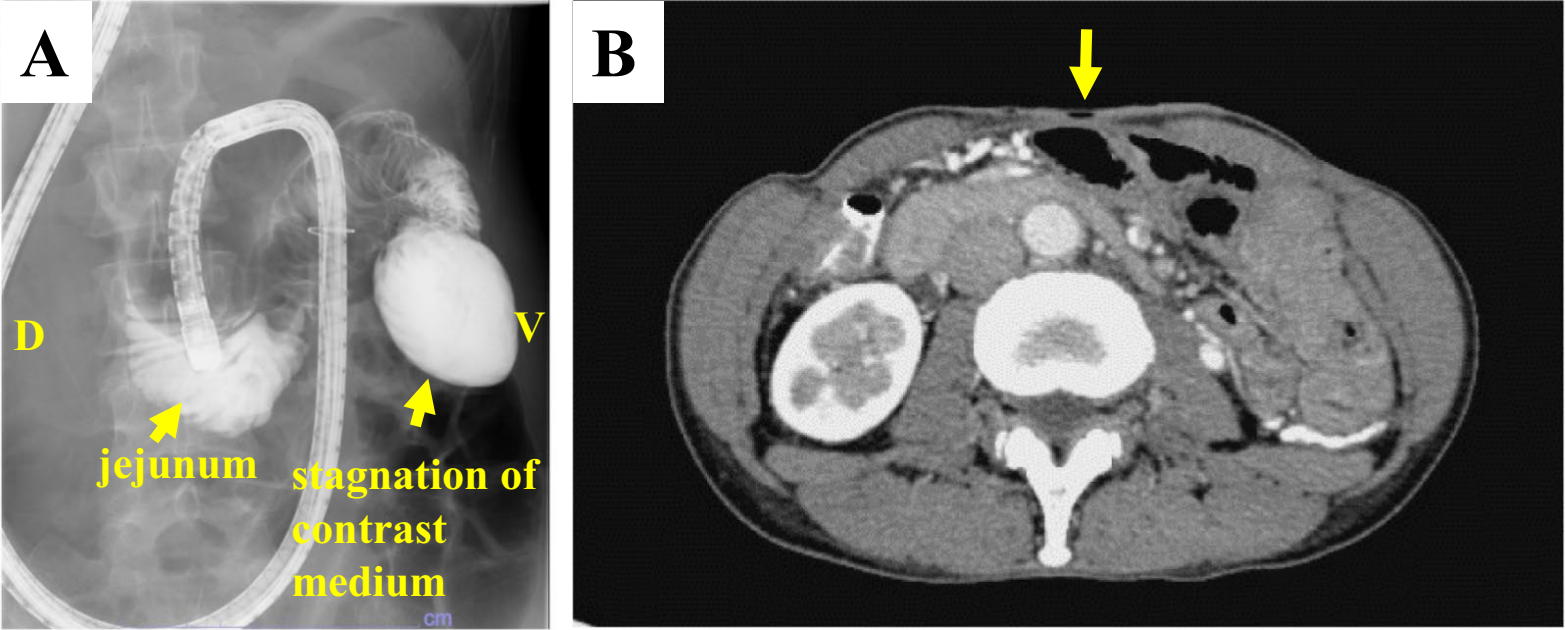
Preoperative imaging examinations are depicted. **A** Enteroclysis revealed stagnation of contrast medium in the jejunum near the ligament of Treitz due to strong flexion. (D: dorsal position V: vertical position). **B** Computed tomography revealing a mass in the dilated bowel, inferior to the fistula. The yellow arrows indicate the presence of an enterocutaneous fistula

Ten-year post-stoma closure, the patient underwent radical surgery. Enteroclysis revealed a strong flexure near the ligament of Treitz (Fig. 2A), and computed tomography (CT) confirmed the presence of a mass of dilated bowel, inferior to the fistula (Fig. 2B). Using a tube guide, a long tube was inserted into the upper jejunum preoperatively for intraoperative evaluation of the bowel passage. After careful preoperative simulation, we planned to perform laparoscopic adhesiolysis around the abdominal wall and mobilization of the pelvic small intestine, thereafter, to convert to an open surgery and evaluated the bowel passage, and subsequently to perform adhesiolysis, resection and anastomosis of the intestines.

**Fig. 3:**
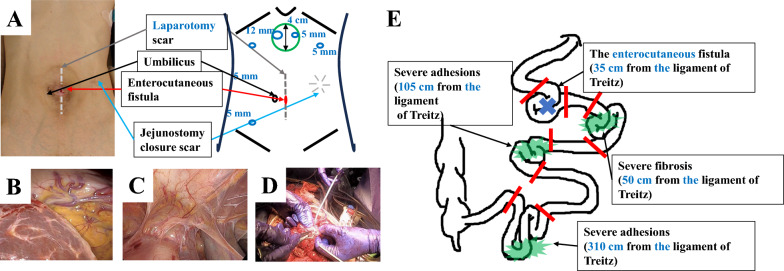
Intraoperative findings are presented. **A** Diagram depicting the skin incision. **B** Upper abdomen has fewer adhesions to the abdominal wall. **C** Lower abdomen has severe adhesions. **D** Dissection between the intestines has been performed under an open conversion. **E** Diagram illustrating the surgical procedure. Four sites of the small intestine have been resected and anastomosed

The patient underwent adhesiolysis and a partial resection of the small intestine at four locations, including the fistula site. The operative time was 634 min, and blood loss was 710 mL. An initial incision was made in the upper abdomen, which was far from the previous wound, and fewer adhesions were anticipated (Fig. 3A). The adhesions in the upper abdomen were not severe (Fig. 3B). Conversely, as postulated, extensive, severe adhesions were found in the lower abdomen (Fig. 3C). The patient underwent laparoscopic dissection of the strong adhesions surrounding the fistula site; dissection of the adhesions, between the abdominal wall and intestine; and mobilization of the pelvic small intestine. Post-open conversion, the adhesions between the intestines were dissected (Fig. 3D). By careful assessment of the entire small intestine inferior to the ligament of Treitz, the ECF was located, 35 cm from the anal aspect of this ligament. The balloon of the long tube was dilated to 15 mm-in-diameter and passed through the terminal ileum, to evaluate for the presence of stenoses and obstructions. The small intestine with difficulty passing through the balloon (four sites in total) were resected and anastomosed (Fig. 3E). A long tube was placed near the anal aspect of the anastomosis. The remaining part of the small intestine was 285 cm-in-length. After confirming blood flow within the remnant bowel, using indocyanine green fluorescence, a skin defect was formed at the fistula site; and the wound was closed.

**Fig. 4:**
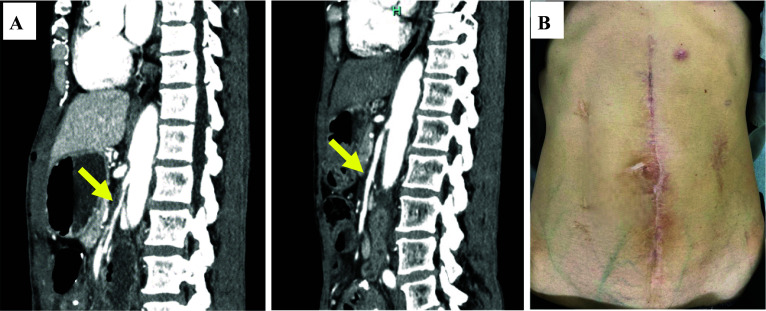
Postoperative course is depicted. **A** Computed tomography (CT) indicating narrowing of the superior mesenteric artery (SMA) with dissociation (left). At 7 months postoperatively, CT reveals that the narrowing of the SMA has resolved (right). **B** Surgical scar is depicted

After confirming the presence of flatus, the long tube was removed. However, repeated episodes of vomiting were observed. The enteroclysis confirmed the absence of organic obstruction of the bowel passage; nonetheless, inadequate peristalsis of the jejunum near the ligament of Treitz and dilatation of the oral aspect of the intestine were revealed. A CT image showed narrowing of the superior mesenteric artery (SMA) with dissociation and intestinal edema (Fig. 4A, left). After a discussion with the interventional radiologists, conservative treatment was continued, because intestinal blood flow was preserved.

However, the patient’s symptoms did not improve, and percutaneous endoscopic gastrostomy (PEG) was performed, to drain fluid in the dilated intestine. Her symptoms gradually resolved with regular drainage and with the use of acotiamide hydrochloride hydrate, mosapride citrate hydrate, and kampo medicines, such as Saireito and Daikenchuto. She could tolerate oral intake 2 months postoperatively and was discharged from the hospital 3 months postoperatively. Seven months postoperatively, the PEG was removed after several days of non-use and in the absence of a recurrence. Moreover, a CT image showed resolution of the SMA narrowing (Fig. 4A, right). Three years postoperatively, the patient progressed without recurrence of the ECF or SBO (Fig. 4B).

## Discussion

In our case of refractory ECF, after combining laparoscopic adhesiolysis with a planned open conversion, we were able to maintain a good surgical field and safely perform the surgery. Moreover, stoma care sufficed, to improve peristomal skin damage, nutritional status, and inflammation control; however, the fistula tract was very short and difficult to close with conservative treatment. This was due to the high output of the jejunal fistula. A long fistula tract, colonic origin, low output from an ECF, and good nutritional status are good predictors of fistula closure [1]. As per previous reports involving a small number of cases, closure rates for the surgical treatment of ECFs range from 50% to 70% [10–14]. However, little improvement in fistula closure rates has been noted over the past few decades [14].

Regarding risk factors for postoperative recurrence, patient factors include diabetes mellitus [1], smoking [1], a high output of > 500 mL/day from an ECF [14], multiple fistulae or abscesses [11, 15], inflammatory bowel disease [16], and small bowel origin [16]. Perioperative factors include operative time [14], blood loss [14], intestinal and fistula resection [17], and the anastomotic method [16]. Fascial closure is effective in preventing recurrence [18, 19]. In our case, the risk factors included a high output of > 500 mL/day from the jejunal fistula, the presence of an abscess, and small intestinal origin. Nevertheless, we were able to close the fistula, by resection of the intestinal tract; while causing fistula and intestinal drainage, using a long tube, to prevent the exertion of intraluminal pressure on the anastomosis.

Standardization of surgical techniques for the treatment of ECFs is difficult, due to the multiple variations thereof. Therefore, careful preoperative simulation of each case is essential. The main causes of compounded challenges encountered in surgery include a history of multiple operations; intra-abdominal adhesions, due to fistula formation; and surrounding infections. In addition, surgery for ECFs bears a high risk of injury to other organs during adhesiolysis. No reports comparing laparoscopic and open approaches to ECFs exists. Moreover, the laparoscopic approach has only been mentioned in a few case reports [18, 20, 21]. Reportedly, however, laparoscopic adhesiolysis is not inferior to open surgery [22, 23]. In particular, when dissecting adhesions from the abdominal wall, laparoscopic surgery includes the advantages of a field of view and applying tension to the dissecting line, by insufflation. This technique can improve the operative field of view and reduce the risk of collateral injury in surgery for an ECF, which frequently involves indistinct tissue boundaries. In our case, severe adhesions were anticipated, and preoperative simulation was performed. The laparoscopic approach was initiated from the epigastric region, where relatively fewer adhesions were anticipated. First, detachment of the adhesions between the abdominal wall and intestine was performed, with a good field of view. Subsequently, a laparotomy and adhesiolysis between the intestines, which required extensive field development and delicate manipulation, were performed under direct vision. This strategy was found to be effective.

Conversely, a limitation of the laparoscopic approach includes the difficulty involved in the selection of the first-port position, in cases where adhesions are postulated to be present throughout the upper and lower abdomen. Largely, cases of ECFs involving the small intestine, have fewer adhesions occupying the upper abdomen. However, preoperatively, port placement should be simulated in patients with a history of upper gastrointestinal surgery.

## Conclusions

Herein, we reported a case of the surgical management of an ECF in a patient with a history of multiple abdominal surgeries. Severe intra-abdominal adhesions were anticipated. Nevertheless, after combining laparoscopic adhesiolysis and a planned open conversion, we maintained a good surgical field and safely perform the surgery. Thus, refractory ECFs require surgical treatment. Furthermore, the surgical approach used in this case may be an option for securing a safe surgical field, while avoiding collateral damage.

## Data Availability

The data sets supporting the findings and inferences of this case report are included in this article.

## Abbreviations

ASPEN: American Society for Parenteral and Enteral Nutrition
CT: Computed tomography
ECF: Enterocutaneous fistula
FELANPE: Federación Latino Americana de Terapia Nutricional, Nutrición Clínica y Metabolismo
PEG: Percutaneous endoscopic gastrostomy
SMA: Superior mesenteric artery
SBO: Small bowel obstruction
